# Cancer-Associated Muscle Wasting—Candidate Mechanisms and Molecular Pathways

**DOI:** 10.3390/ijms21239268

**Published:** 2020-12-04

**Authors:** Victoria S. Armstrong, Liam W. Fitzgerald, Oliver F. Bathe

**Affiliations:** 1 Arnie Charbonneau Cancer Research Institute, Cumming School of Medicine, University of Calgary, Calgary, AB T2N 4Z6, Canada; victoria.armstrong@ucalgary.ca (V.S.A.); liam.fitzgerald1@ucalgary.ca (L.W.F.); 2 Department of Medical Sciences, University of Calgary, Calgary, AB T2N 4Z6, Canada; 3 Departments of Surgery and Oncology, University of Calgary, Calgary, AB T2N 4Z6, Canada

**Keywords:** sarcopenia, muscle wasting, cancer, pathophysiology, mediators, tumor, tumor-derived

## Abstract

Excessive muscle loss is commonly observed in cancer patients and its association with poor prognosis has been well-established. Cancer-associated sarcopenia differs from age-related wasting in that it is not responsive to nutritional intervention and exercise. This is related to its unique pathogenesis, a result of diverse and interconnected mechanisms including inflammation, disordered metabolism, proteolysis and autophagy. There is a growing body of evidence that suggests that the tumor is the driver of muscle wasting by its elaboration of mediators that influence each of these pro-sarcopenic pathways. In this review, evidence for these tumor-derived factors and putative mechanisms for inducing muscle wasting will be reviewed. Potential targets for future research and therapeutic interventions will also be reviewed.

## 1. Introduction

In cancer, loss of muscle mass most commonly occurs in the context of the cancer cachexia syndrome, the involuntary loss of skeletal muscle mass, with or without accompanying adipose tissue wasting [1]. Muscle wasting in association with loss of strength and function is referred to as sarcopenia and this is also common in the setting of cancer. Importantly, muscle wasting may still occur despite preservation of substantial adipose tissue, as seen in sarcopenic obese individuals [2]. Low muscle mass is present in as many as 80% of patients with pancreatic and gastric cancers, 50% with colorectal and lung cancers and 40% with breast cancers [3]. It is more common in inpatients (51%) compared to outpatients (22%); terminal cancers (26%) compared to curable cancers (17%); and metastatic (25%) vs. locoregional cancers (19%) [4]. Moreover, as individuals are followed longitudinally as disease progresses, muscle wasting becomes more severe [5]. The increased prevalence of muscle wasting coincidental with cancer, its variability between cancer types and its progression with advanced disease suggests that it represents a distinct host response to the tumor itself. Understanding the mechanisms of this host response is imperative to devising a strategy to reverse muscle wasting and to improve its functional sequelae.

Muscle loss has significant effects on patient health and quality of life. It is accompanied by weakness and frailty, leading to loss of independence. It is associated with impaired immunity and susceptibility to infections. Sarcopenic patients have a diminished capability to tolerate treatments, including surgery and chemotherapy. Importantly, numerous studies have demonstrated shorter survival in individuals with measurable muscle wasting [6,7,8]. Given the clinical implications including impaired quality of life, it is imperative that new, better therapies be devised. That will require an improved understanding of the pathophysiological drivers of muscle wasting.

Cancer-associated muscle wasting differs substantially from the sarcopenia that frequently accompanies aging (“primary sarcopenia”). Primary sarcopenia can be effectively treated with exercise and dietary intervention. The muscle wasting associated with cancer, on the other hand, is intractable and cannot be completely reversed with enhanced nutritional support [1]. The pathophysiologic mechanisms appear to differ and understanding those differences will instruct therapeutic interventions. Our hypothesis is that cancer-associated muscle wasting is driven by tumor-derived circulating factors, including mediators of metabolism and inflammation. The purpose of this review is to survey what is known about these tumor-derived pro-sarcopenic mediators and to explore potential strategies that could be used to treat cancer-associated muscle wasting.

## 2. Pathophysiological Features of Muscle Wasting

**Figure 1:**
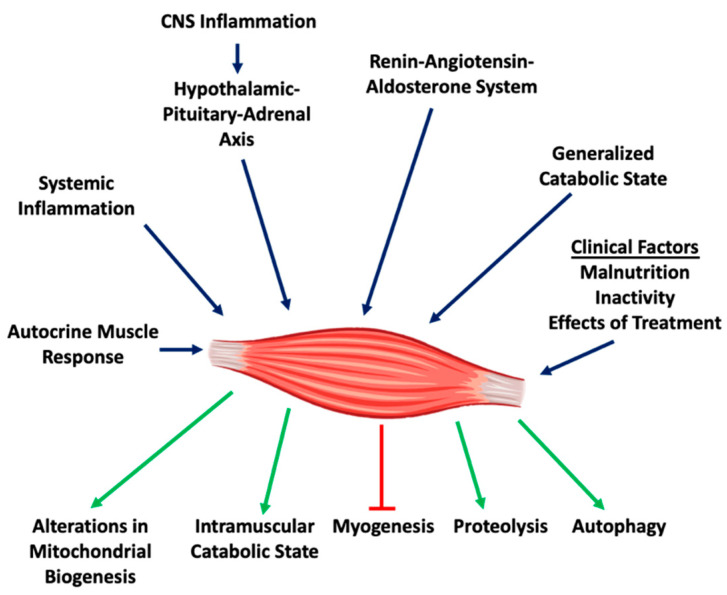
General mechanisms contributing to the pathogenesis of muscle wasting.

Past studies have highlighted a variety of underlying mechanisms and pathways that contribute to cancer cachexia and cancer-related muscle wasting. Perhaps the most prominent of features are systemic inflammation and metabolic derangements. Other attributes of cancer-associated muscle wasting include enhanced autophagy, inhibition of myoblast differentiation and derangements in the renin-angiotensin system which all contribute to muscle degradation (Figure 1). As one might expect, these processes are interconnected.

### 2.1. Systemic Inflammation

Inflammation is a known concurrence of cancer and cachexia [9,10]. Because it is not universally present in individuals with cancer, it cannot be considered a hallmark of cancer. However, once manifested, it is known to encourage tumor growth and progression, enhancing the hallmarks that typify cancer [11]. Indeed, systemic inflammation has broadly been reported to be associated with poor survival outcomes in a number of cancers [12,13,14,15,16]. Moreover, systemic inflammation has been linked with weight loss and muscle wasting [17].

C-reactive protein (CRP) is an acute phase reactive protein that rises in response to acute inflammation and tissue damage. Elevated levels of CRP are also observed in some cancer patients [18]. High CRP levels have been associated with poor survival in a wide variety of cancers [14,19,20,21,22,23,24,25]. Elevated CRP levels have also been reported in patients with cachexia and muscle wasting [26,27,28].

CRP is under the transcriptional control of various cytokines but the principal stimulant of CRP transcription is interleukin-6 (IL-6) [29]. In patients with cancer, CRP levels are well known to correlate with IL-6 levels [14,30]. In mice, IL-6 administration induces cachexia [31] and IL-6 blockade reverses muscle loss in cancer cachexia models [32,33,34]. IL-6 levels in particular have been shown to correlate with weight loss and reduced survival in cancer patients [35,36].

Interleukin-1β (IL-1β) also appears to play a central role in muscle wasting. Normal myogenic cells constitutively express IL-1β and exposure to exogenous IL-1β induces myogenic cell apoptosis [37]. IL-1β also induces IL-6 production in muscle [38], further perpetuating the local intramuscular proinflammatory milieu.

Interleukin-8 (IL-8) has also been described in association with muscle wasting. Increased serum and tumor tissue levels of IL-8 have been described in sarcopenic pancreatic cancer patients [39]. In gastric cancer patients, a polymorphism of IL-8 was associated with cachexia [40]. IL-8 is sufficient to cause myotube atrophy in vitro; in vivo, it can induce atrophy of the diaphragm muscle in mice [41]. A microarray experiment suggested that upregulation of ubiquitin-proteasome pathways may be involved. On the other hand, in rat skeletal muscle cells, IL-8 also increases myocilin expression, Akt/protein kinase B phosphorylation and reduced forkhead box O3 (FoxO3) levels—evidence that IL-8 is actually an anti-catabolic factor for skeletal muscle [42]. Given these conflicting findings, more work will need to be done to understand the role of IL-8 in the pathogenesis of muscle wasting.

Signaling through the tumor necrosis factor (TNF) superfamily ligand TWEAK (TNF-like weak inducer of apoptosis) and its receptor Fn14 (fibroblast growth factor-inducible 14) has profound effects on the state of skeletal muscle, particularly in response to acute and chronic tissue injury. TWEAK is constitutively expressed widely in various tissues; Fn14 expression is induced by pro-inflammatory cytokines released as a result of injury. TWEAK induces muscle wasting in mice [43]. Age-related sarcopenia is associated with elevated circulating TWEAK levels that are normalized with an intensive lifestyle intervention program [44] but few data are available in the context of cancer-associated muscle wasting.

Tumor necrosis factor-α (TNFα) is produced by various myeloid cells, especially macrophages and it is rapidly increased following tissue injury. TNFα is also produced by satellite cells, myogenic stem cells that are essential for muscle regeneration and growth [45]. Exogenous TNFα in mice diminishes muscle mass and regenerative capacity [46,47]. TNFα derived from myeloid cells and TNFα intrinsic to muscle both appear to contribute to age-related sarcopenia [45].

While there are certainly ample preclinical data that demonstrate the capability of proinflammatory factors to induce cachexia and muscle loss, clinical observations make it far from clear whether a proinflammatory state is a sine qua non of muscle wasting in general or even cancer-associated muscle wasting. In a study of patients with advanced cancer, Scheede-Bergdahl et al., measured CRP and pro-inflammatory cytokines (IL-6, IL-1β, IL-8 and TNFα), correlating them with various clinical features of cachexia [30]. High CRP levels were generally associated with elevations in each of the proinflammatory cytokines. Interestingly, IL-1β was the only cytokine independently associated with weight loss. High IL-1β and TNFα were significantly associated with muscle wasting; and there was a trend toward a positive association between muscle wasting and IL-6 and IL-8 levels. Talbert and coworkers reported that, in a group of patients with resectable pancreatic cancer, there was no significant association of IL-6, IL-1β, interferon-γ (IFN-γ) and tumor necrosis factor with cachexia [48]. In a meta-analysis comparing pro-inflammatory mediators in 3072 individuals with muscle wasting and 8177 individuals without muscle wasting, patients with low muscle mass had significantly higher levels of CRP but did not have increased IL-6 and TNFα levels [26]. However, it is unclear from the analysis how many of the subjects included in the study had cancers.

These clinical studies bring to light some inherent challenges in the study of disease-related changes in body composition. First, muscle wasting must be separately studied from the syndrome of cachexia (which may indeed represent a heterogeneous group of states). Second, cancer-associated muscle wasting may have an entirely different pathogenesis than sarcopenia secondary to aging, inactivity and other chronic disease states. Third, in most human studies, circulating factors have been the focus of study, making it difficult to ascertain the source of any inflammatory response. Finally and importantly, it must be acknowledged that not all patients with cancer-associated muscle wasting have objective evidence of systemic inflammation. Therefore, other factors and pathogenic mechanisms must be considered.

### 2.2. Central Nervous System (CNS) Inflammation

In addition to its local effects in muscle, IL-1β can exert its effects on the hypothalamic-pituitary-adrenal axis. Braun and collaborators demonstrated that tumor-bearing mice have markedly increased hypothalamic levels of IL-1β [49]. Simultaneously, muscle loss occurs in conjunction with the induction of skeletal muscle protein degradation pathways. Direct intracerebroventricular injection of IL-1β has similar atrophic effects on muscle and this is not attributable to leakage of IL-1β into the systemic circulation or to the induction of high levels of systemic IL-6. Rather, adrenalectomy ablates the effect and glucocorticoids are necessary for the atrophic effects on muscle.

### 2.3. Generalized and Intramuscular Catabolic States

The weight loss that often accompanies cancer is multifactorial. Nutritional deficiencies, reduced activity levels and miscellaneous treatment effects may contribute. Increased resting energy expenditure (REE) has been documented in pancreatic cancers, lung cancers and sarcomas [50,51,52]. A contributory factor may be the tumor itself, which characteristically has a high rate of glycolysis as well as other accelerated metabolic processes to support high cell turnover. Indeed, REE tends to be higher in more advanced cancer stages when the tumor burden is greater [53].

A metabolic state biased toward catabolism may also result from the neuroendocrine and inflammatory response generally related to chronic illness. In chronically ill patients and individuals in a pro-inflammatory state, there is dysregulation in the hypothalamic-anterior pituitary-peripheral endocrine axis: alterations in growth hormone, thyroid hormone, sex steroids and cortisol occur. Perhaps most pertinent to the pathogenesis of muscle wasting, chronic pro-inflammatory states are associated with depressed levels of insulin-like growth factor-1 (IGF-1) and IGF binding protein-3 (IGFBP-3), leading to a state of peripheral resistance to growth hormone and impaired anabolism [54]. In addition to these neuroendocrine events, in cancer patients, inflammation is in general associated with a high REE [55,56,57], although the exact mechanisms for this is unclear.

In addition to a generally catabolic host state, metabolic changes appear specifically at the level of the muscle. High cortisol levels stimulate glycogenolysis and proteolysis in muscle. Inflammatory stimuli including IFN-γ and TNFα result in release of IL-6 from myocytes [58] and subsequent alterations in mitochondrial biogenesis may ensue. In myotubules, IL-6 can cause repression of peroxisome proliferator-activated receptor gamma coactivator 1-alpha (PGC1α), the master regulator of mitochondrial biogenesis [59]. TWEAK also impairs oxidative metabolism in muscle, likely by decreasing PGC1α expression and activation of NF-κB [60].

Alterations in skeletal muscle mitochondria have been documented in the context of cancer. In patients with muscle wasting associated with gastrointestinal malignancies, muscle mitochondria have distinct morphological and functional features including increased mitochondrial volume and dysfunctional autophagy in association with increased skeletal muscle apoptosis [61]. Cancer-associated muscle wasting is associated with decreased expression PGC1α, which may be mediated by IL-6 [59,62]. Perturbations in skeletal muscle mitochondrial function such as diminished oxidative capacity and reductions in cytochrome C oxidase and ATP synthase content have been observed in the context of tumor [63]. Indeed, even before muscle atrophy becomes apparent, reductions in mitochondrial content and oxidative capacity have been observed in murine models of cancer cachexia [64].

### 2.4. Increased Muscle Protein Degradation

Muscle proteolysis is accelerated in virtually every form of muscle wasting. Ultimately, muscle protein catabolism involves upregulated expression of two muscle-specific E3 ubiquitin ligases, muscle ring finger 1 (MuRF1) and muscle atrophy F-box (MAFBx)/atrogin-1. In general, MuRF1 and MAFBx bind selective substrates for ubiquitination and subsequent degradation by the 26S proteasome [65]. MuRF1 and MAFBx gene expression is upregulated as a result of diverse stimuli, including physical inactivity, malnutrition and exposure to catabolic mediators such as cytokines and glucocorticoids.

Activation of the IGF-1/phosphatidylinositol 3-kinase (PI3K)/Akt pathway protects against muscle atrophy by preventing induction of MuRF1 and MAFBx [66], as well as maintaining stem cells essential for muscle regeneration [67]. Akt-induces inactivation of FOXO transcription factors, which is essential to these processes. Similarly, insulin-like growth factor-2 (IGF-2) stimulates myogenesis by binding and activating the IGF-1 receptor [68]. Therefore, anything that restrains binding of insulin-like growth factors to the IGF-1 receptor such as insulin-like growth factor binding proteins (IGFBPs) could potentially induce muscle atrophy [69,70].

Inflammatory mediators are also integral to the anabolic and catabolic processes in muscle. NF-κB signaling secondary to proinflammatory cytokines including TWEAK induces degradation of muscle proteins such as myosin heavy chain, as well as activation of the ubiquitin proteasome pathway [43,71,72,73]. TNFα -induced muscle wasting is mediated by the induction of MuRF1 [74]. In addition to systemic inflammation, hypothalamic inflammation can also result in the activation of MuRF1 and MAFbx [49]. In the context of tumor, IL-1β appears to be the most important inflammatory mediator at the level of the hypothalamus and glucocorticoids are essential for the effects on protein degradation at the level of the muscle.

### 2.5. Inhibition of Myoblast Differentiation

Ligands binding to the activin receptor type IIB (ActRIIB) appear to be central to the muscle wasting seen in association with cancer. ActRIIB is a high affinity transmembrane receptor for a subset of transforming growth factor-β (TGFβ) family ligands including activin A and myostatin. Binding of activin A to ActRIIB results in the formation of a heteromeric receptor complex with the recruitment of a type I receptor, followed by signaling through Smad pathways. Antagonism of ActRIIB reverses skeletal muscle loss and heart atrophy in multiple cancer cachexia models by inhibiting proteolysis and stimulating muscle stem cell growth [75].

Myostatin (also known as growth differentiation factor 8, GDF8) and activin A are negative regulators of muscle size, inhibiting muscle cell differentiation. Myostatin inhibits muscle differentiation and also inhibits Akt-induced protein synthesis [76]. Myostatin is released by myocytes and acts in an autocrine fashion to inhibit myogenesis. Its expression in muscle is upregulated in animals with tumor-induced cachexia [75]. Administration of exogenous myostatin induces profound muscle and fat loss [77]. Activin A, in addition to its role in regulation of muscle mass, has diverse functions including regulation of the menstrual cycle and regulation of wound healing [78]. Elevated circulating levels of activin A are sufficient to cause muscle loss in mice [79]. The most important physiologic inhibitors of the bioactivity of myostatin and activin A are follistatin and follistatin-like 3 (FSTL3) [80]. As these proteins are derived from the same tissues, they may represent an autocrine control mechanism based on a negative feedback loop.

Increased circulating levels of activin A have been reported in cancer patients [81,82]. In contrast, circulating myostatin levels have been reported to be decreased [83]. Interestingly, Loumaye and collaborators found that activin A levels were elevated in cachectic patients in comparison to non-cachectic patients but myostatin levels were decreased in association with cachexia [84]. It is possible that the reduced myostatin levels in these studies represent a homeostatic response to high activin A levels, as a protective measure to preserve muscle mass.

The TWEAK-Fn14 axis also plays a role in myocyte differentiation. Fn14 is constitutively expressed in muscle progenitor cells including myoblasts and satellite cells [85]. In conditions of chronic inflammation and tissue injury, engagement with TWEAK stimulates NF-κB signaling pathways in myoblasts, inhibiting myoblast differentiation [85,86]. TWEAK also inhibits elaboration of miRNAs that are essential for muscle progenitor cell proliferation and differentiation [87]. Other proinflammatory factors additionally play a potential role in myocyte viability. TNFα, for example, diminishes myogenic differentiation through NF-κB transcriptional activation and destabilization of myoblast determination protein 1 (MyoD) [88,89].

### 2.6. Enhanced Autophagy

Autophagy represents a normal physiological process where cells break down unnecessary or dysfunctional cellular organelles. The catabolites generated by this degradation are recycled to be utilized by newer, healthier cells. In the context of cancer, where the high proliferative rate of tumor cells produces a nutrient-limited microenvironment, autophagy can be induced in normal cells, generating nutrients beneficial to tumor cells.

Autophagy also plays an important role in the homeostasis of muscle. It is essential for the maintenance of the regenerative capacity of Pax7-expressing muscle stem cells (satellite cells) [90]. It protects against muscle atrophy, maintains intramuscular protein quality and preserves muscle function [91]. During aging, there is a decline in autophagy in muscle which can be inhibited by resistance exercise [92]. The transcription factor forkhead box O (FoxO) plays a central role in muscle atrophy, inducing expression of genes that increase autophagic degradation in muscle [73,93]. Mechanistic target of rapamycin complex 1 (mTORC1) signaling also plays a central role; sustained mTORC1 signaling, leading to autophagy inhibition, is seen in the muscle of aged mice [94].

In contrast, in pathological muscle wasting, induction of autophagy leads to muscle wasting. In mice, autophagy can be induced by causing muscle degradation through several mechanisms including glucocorticoid-induced atrophy, caloric restriction and cancer cachexia [95]. In cancer bearing mice, stimulation of muscle autophagy exacerbates muscle atrophy and this is thought to be related to activation of mitophagy and impairment of mitochondrial function [96]. Others have similarly linked cancer-associated muscle wasting with accelerated autophagy [97]. He et al., observed that sera from tumor bearing mice as well as pancreatic cancer patients causes NF-κB-dependent overexpression of the self-renewing factor Pax7 by (non-satellite) muscle progenitor cells, which drives muscle wasting [98]. Both IL-6 and activin A, prominent mediators of cancer-associated muscle wasting, can drive autophagy in muscle cells [97,99]. In all, the data suggest that cancer-associated muscle wasting differs markedly from age-related sarcopenia in that autophagy accelerates it.

### 2.7. The Renin-Angiotensin Pathway

The renin–angiotensin–aldosterone system (RAAS) is better known for its role in regulation of fluid-electrolyte balance, systemic vascular resistance and blood pressure. In this cascade, the angiotensin-converting enzyme (ACE)/angiotensin II (Ang II)/angiotensin II receptor type 1 (AT1) axis induces physiological effects, such as vasoconstriction, cell proliferation and fibrosis while the ACE2/Angiotensin 1–7 (Ang-(1–7))/Mas receptor axis protects against end-organ damage. The RAAS also plays an important role in muscle homeostasis, where Ang II-associated muscle breakdown is counteracted by Ang (1–7), which promotes protein synthesis. In cancer, overexpression of AT1 and increased circulating Ang II have been associated with more aggressive tumors [100].

Deranged RAAS may also play a role in muscle wasting. The data are most convincing in the context of cardiac cachexia but RAAS may also play a role in cancer-associated muscle wasting. In a murine model of adenocarcinoma, Ang II was shown to reduce muscle protein synthesis [101]. Ang II also has a number of other adverse effects on muscle mass. Ang II induces muscle atrophy by increasing atrogin-1 and MuRF1 expression [102,103]. Reactive oxygen species (ROS) are produced when circulating Ang II binds to AT1 on skeletal muscle cells and can subsequently induce atrogin-1 expression [104]. In animal models of muscle wasting, Ang II disrupts IGF-1 signaling, resulting in net protein catabolism [105,106,107]) and Ang II detrimentally affects muscle metabolism and energy stores [107].

## 3. Tumor-Derived Mediators of Muscle Wasting

While some of the pathophysiologic features of cancer-associated muscle wasting are well recognized, what is less well understood is what initiates and drives it. The preponderance of studies in the field have focused on circulating factors and their effects on muscle; studies so designed shed little light on the singularity event that induced muscle loss. It seems intuitive that the tumor itself plays a central role, perhaps driving muscle loss.

### 3.1. Evidence for the Role of Tumor on Muscle Wasting

What is the evidence that tumor drives muscle wasting? Afterall, muscle loss and sarcopenia can emerge in the context of diverse chronic illnesses. Inactivity, malnutrition and miscellaneous effects from therapy could each contribute to the muscle wasting that accompanies cancer.

**Figure 2:**
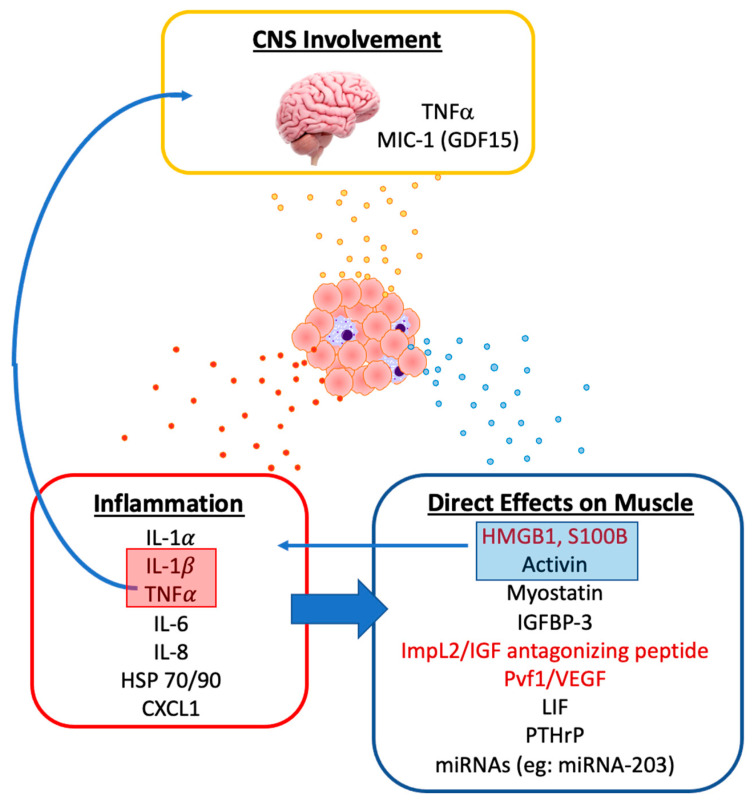
Tumor-derived factors have been described that are pro-inflammatory in nature, target the central nervous system (CNS) and directly target muscle. Mediators in red are also known to encourage tumor growth.

The first line of evidence is preclinical. A number of in vitro and in vivo studies—described below—have demonstrated that tumor-derived factors induce muscle wasting. Clinical observations also support that tumor plays an active role in muscle wasting. Not only is muscle wasting more common in cancer patients but some cancers such as pancreatic cancer and lung cancer are associated with disproportionally high incidences of muscle wasting. This suggests that their biology has special features that induce muscle loss. Muscle wasting becomes more prevalent with a greater burden of disease, which may be related to elaboration of more of the tumor-derived mediators that drive muscle loss. What would add to the clinical aggregation of proof is a study that demonstrates the reversal of muscle wasting with removal of tumor, although this has proven complicated to study because surgery itself has adverse effects on muscle mass, as do other cancer treatments. Therefore, at present, we are reliant on observations of tumor-derived mediators that have the capability to drive muscle wasting (Figure 2).

### 3.2. Tumor-Derived Pro-Inflammatory Mediators

The tumor secretome differs from the secretome of normal tissues and it is diverse; it includes factors that are known to induce muscle loss [108]. To identify potentially secreted biomarkers in non-small cell lung cancer patients that were associated with muscle wasting, Cury et al., identified differentially expressed genes in tumor from patients with low muscularity [109]. Low muscularity was associated with 75 overexpressed genes including IL-6, colony stimulating factor 3 (CSF3) and IL-8. IL-8 was consistently found to be a bad prognostic factor in validation sets. IL-8 was shown to induce in vitro myotube atrophy. Hou and coworkers have shown that tumor expression of IL-8 is also associated with muscle loss in pancreatic cancer [39]. Further investigation will be required to identify which other factors that are differentially abundant in tumors are important drivers of muscle wasting.

Much attention has been paid to the capability of tumor to promote inflammation, thought to be a primary driver of cancer-induced muscle wasting. This has been thoroughly reviewed [10,110]). The question is whether there is some characteristic of tumor that provokes elaboration of pro-inflammatory mediators from tumor. Johnson and coworkers demonstrated that in breast, head and neck, lung, colorectal and stomach cancer, the expression of pro-inflammatory cytokines (IL-1α, IL-1β, IL-6, IL-8 and TNFα positively correlates with expression of Fn14, the cognate receptor for TWEAK [111]. In mice bearing Fn14-expressing tumors, antibodies against Fn14 markedly reduce tumor-induced weight loss and extended lifespan. Zhang et al., reported that a number of cachexia-inducing tumor cells constitutively release heat shock proteins 70 and 90 (Hsp 70/90) in association with extracellular vesicles [112]. Muscle catabolism is mediated by toll-like receptor 4 (TLR4) activation, causing a systemic pro-inflammatory state. Inhibition of Hsp 70/90 expression abrogates tumor-induced muscle catabolism. Additionally, in an orthotopic pancreatic carcinoma mouse model, zinc transporter 4 (ZIP4) was shown to induce the secretion of HSP 70/90 to induce p38 mitogen-activated protein kinase (MAPK)-mediated muscle wasting [113].

Activin A appears to have an important role in stimulating an inflammatory response at the level of the tumor. Several human tumor cell lines secrete activin A and myostatin [75,97]. In patients with lung cancer and colorectal cancer, activin A levels correlate with inflammatory markers such as CRP [84]. CRP levels are well known to correlate with IL-6 [14,30], a pro-cachectic pro-inflammatory cytokine known to be elaborated by some tumor cells [99,114]. In an ovarian cancer cell line known to cause rapid development of cachexia, Pettersen et al., demonstrated that activin elaborated by tumor cells is a required autocrine signal for IL-6 secretion [97]. Interestingly, autophagy of non-cancerous cells can be induced by IL-6 but not activin A. These data suggest that any role in autophagy that has been attributed to activin A may actually be mediated by IL-6. Indeed, administration of ActRII neutralizing antibodies to tumor-bearing mice effectively reduces circulating IL-6 levels and reversed muscle loss and other manifestations of cachexia.

Any tumor consists of a diverse population of cells. In addition to cancer cells, the stroma consists of a mélange of fibroblasts, endothelial cells and immune cells as well as other cell types. These non-cancerous cells may also significantly contribute to pro-sarcopenic mediators that emanate from a tumor. One example of this was reported by Callaway and colleagues, who studied factors that were secreted from human pancreatic cancer cells, tumor-associated stromal cells and their co-culture [41]. Conditioned media from co-cultures induce greater myotube atrophy than conditioned media from either cell type alone. Pancreatic cancer cells stimulate the release of IL-8, IL-6 and interferon gamma-induced protein 10 (IP-10) from stromal cells and IL-8 induces skeletal muscle atrophy in vivo. In all, these data demonstrate that the cross-talk between cancer cells and tumor-associated stromal cells is integral to the secreted signals that emanate from tumor.

Hogan et al., reported that lung cancer cell lines produce a number of factors that suppress myoblast differentiation, including IGFBP-3, C-X-C motif chemokine ligand 1 (CXCL1) and C-C motif chemokine ligand 2 (CCL2) [115]. The most differentially and highly expressed cytokine in their cell line experiments was CXCL1, which was also elevated in the serum and muscle of tumor bearing mice. In vivo, CXCL1 impairs muscle regeneration. Administration of CXCL1 stimulates intramuscular recruitment of neutrophils and M2 macrophages, which are known to participate in tissue repair and remodeling [116]. Others have also demonstrated tumor-derived secretion of CXCL1, which has pleiotropic effects in the tumor microenvironment [117,118,119,120].

### 3.3. Tumor-Derived Mediators of Metabolism, Protein Catabolism and Myogenesis

Activation of the IGF-1/PI3K/Akt pathway induces protein anabolism in conjunction with blocking protein degradation induced by the FoxO family [121]. Therefore, when the IGF-1/PI3K/Akt pathway is inhibited, more enhanced muscle catabolism can occur. Factors that bind IGF effectively reduce their bioavailability and therefore inhibit the IGF signaling pathway. Specifically, IGFBP-3, the most abundant of IGFBPs in serum, is significantly overexpressed in pancreatic cancers [69]. IGFBP-3 inhibits myogenesis and induces proteolysis but does not appear to have any effects on the viability and proliferative capacity of the tumor cells themselves [69].

Song and colleagues used a fly model of tumor induced organ wasting to identify pathways operative in both tumor and host tissues [122]. Induction of active oncogene yorkie, the homolog of human YAP1, causes muscle protein degradation and impairment of myofiber integrity in association with muscle dysfunction. The effect is related to activated MEK (mitogen-activated protein kinase) signaling in host tissues. MEK signaling promotes muscle atrophy by modulating ubiquitin-dependent protein degradation [123]. Autonomous MEK activation also supports tumor growth [124]. Tumor-derived ligands considered to be contributory include ImpL2 (an IGF antagonizing peptide) and Pvf1 (a homolog to mammalian vascular endothelial growth factor; VEGF) [122,125]). While the fly model is quite disparate from mammalian models, the observations are intriguing and further studies on the role of MEK signaling should be pursued.

Another tumor-derived cytokine, leukemia inhibitory factor (LIF), has been associated with MEK activation and other muscle catabolic processes [126]. In the murine C26 colon carcinoma model, LIF emerged as an important tumor-derived factor that induced atrophy in myotubules. In vivo, LIF can induce reductions in muscle mass and myofiber size but fat mass is unaffected [127]. Increased serum levels of IL-6 and G-CSF (also known as CSF3) appear as a consequence of elevated levels of LIF but these cytokines are not contributory factors to the muscle wasting seen in this model.

### 3.4. Other Tumor-Derived Factors Associated with Muscle Wasting

The RAGE (receptor for advanced glycation end-products) and its ligands, high mobility group box 1 (HMGB) and S100B have documented roles in skeletal muscle and in tumor. HMGB1 and S100B are released upon cell injury and necrosis [128,129]. Engagement of HMGB1 and S100B with RAGE in neighboring cells activates elements of the innate and adaptive immune system [130]. In muscle, S100B, HMGB1 and RAGE appear to be involved with myogenesis and muscle regeneration [131,132]. Upregulation of RAGE has been documented in some cancers [133,134] but not all [135]. Cancer-related elevations in S100B and HMGB1 levels have also been reported [136,137]. HMGB1 overexpression is associated with a worse prognosis [138,139,140]. Indeed, RAGE ligands have growth factor functions that encourage proliferation, invasion and metastasis of cancer cells [128,141]. In murine cancer models, HMGB1 and S100B induce muscle wasting and this is associated with elaboration of IL-1β and IL-6 [142,143]. In all, these data demonstrate that tumor-derived HMGB1-S100B/RAGE signaling adversely affects muscle mass as well as tumor biology, possibly also affecting inflammatory and immune events.

Kir et al. [144] demonstrated, in a Lewis lung carcinoma model of cancer cachexia, that tumor-derived parathyroid hormone-related protein (PTHrP) drives genes involved in thermogenesis (“browning”) in adipose tissues. While PTHrP does not directly affect muscle mass, it does markedly exacerbate skeletal muscle wasting and muscle dysfunction that occurs in the presence of tumor. This suggests that PTHrP acts in concert with other tumor-derived factors to effect muscle loss. Clinically, detectable PTHrP in the circulation is associated with lower lean body mass.

MicroRNAs represent another class of molecules that may be involved in muscle wasting. Hur et al., demonstrated elevated levels of miR-203 levels in CRC metastases [145]. Elevated miR-203 in the serum of colorectal cancer patients is associated with muscle wasting [146]. Transfection of muscle cells with miR-203 inhibits proliferation and promotes apoptosis. The target for miR-203 is BIRC5 (survivin), an inhibitor of apoptosis. A number of microRNAs preferentially expressed in skeletal muscle have been described and these have functional effects on myogenesis and regeneration [147]. It is possible that tumor may have the capability to produce more of such microRNAs, which requires further investigation.

Finally, tumor may be the source of factors that induce anorexia which may exacerbate the effects of factors that directly act on muscle. Borner and coworkers described a rat hepatoma model in which animals became progressively more anorexic as tumor burden increased; weight loss and muscle wasting ensued [148]. An experimental lesion in the area postrema of the brainstem lesion ablates the anorexia and the accompanying loss of muscle. Lesions in this area of the brainstem also attenuate TNFα-induced anorexia in rats (8). In the rat hepatoma model, circulating levels of IL-1β, IFN-γ, IL-6 and TNFα are not increased. Rather tumor growth and anorexia correlate with circulating levels of macrophage inhibitory cytokine-1 (MIC-1)—also known as growth differentiation factor 15 (GDF15). MIC-1 is overexpressed in human tumors [149,150]. Borner et al., speculated that MIC-1 induced anorexia via a direct effect on the area postrema which lacks a functional blood-brain barrier and is therefore exposed to circulating mediators [148].

## 4. Opportunities for Clinical Benefit

Evidence is accumulating that tumors are the source of mediators that cause loss of muscle mass. It is possible that tumor-derived mediators also incite other changes in body composition such as myosteatosis. A comprehensive appreciation of the range of mediators will shed light on underlying mechanisms that can be specifically targeted for therapeutic purposes.

Drugs that are already available can be repurposed for the treatment of muscle wasting and sarcopenia. Angiotensin receptor blockers (angiotensin II inhibitors) are in use to treat hypertension. MEK inhibitors such as trametinib, cobimetinib and binimetanib are approved for the treatment of melanoma. Docosahexaenoic acid (DHA) modulates the ubiquitin-proteasome and the autophagy-lysosome systems. DHA can delay muscle wasting by stimulating oxidative stress and inhibiting proteasomal degradation of muscle proteins [151]. Novel therapeutics can also be considered. Cases have been reported describing a favorable response to toclizumab, an antibody to IL-6 receptor [152,153]. Finally, there may be strategies that simultaneously inhibit tumor growth and ameliorate muscle wasting. Examples include MEK inhibitors, poly(adenosine diphosphate-ribose) polymerase (PARP) inhibitors, tyrosine kinase/platelet-derived growth factor receptor (PDGFR) inhibitor imatinib [154,155,156,157]. Inhibition of the ActRIIB receptor may also provide dual benefit. Blockade of ActRIIB reduces tumor size and metastasis in mice bearing the Lewis lung carcinoma [158,159]; an activin receptor-like kinase inhibitor, SB-431542, has been developed but has not yet entered clinical use [160].

Given the range of mediators secreted by tumors in various reports, it is entirely possible that no single drug can be used to prevent or treat cancer-associated muscle wasting. Pro-sarcopenic factors may differ between tumor types and even between individuals. If this proves to be the case, then the treatment of cancer-associated cachexia may become as complex as the treatment of the cancer itself, requiring an individualized approach.

One consideration is that chemotherapy and some of the more recently introduced targeted agents used to treat the cancer may incite muscle wasting. Cytotoxic agents such as irinotecan, cisplatin, doxorubicin and etoposide can provoke activation of NF-κB and the ubiquitin proteasome pathway [161]. Targeted agents known to induce muscle loss include sorafenib [162] and mTOR inhibitors such as everolimus [163]. Some of these effects may be ameliorated by vitamin D replacement, DHA, testosterone, selective androgen receptor modulators and ghrelin [161], although larger trials will be required to refine the indications for these treatments.

Finally, non-pharmacological treatments require further consideration. In recent years, many studies have demonstrated the benefits of exercise in cancer patients, even those with metastatic disease [164,165]. Unfortunately, few studies have examined the benefits of exercise specifically in cancer cachexia [166]. One challenge in understanding data related to the benefits of exercise in the context of cancer is the inherent difficulty in distinguishing muscle wasting as a result of tumor (“cachexia”) and muscle wasting secondary to age, malnutrition, disuse and treatment effects [167]. Advanced diagnostics will be important in this regard. Another challenge is a practical one. Instituting an exercise program that is appropriate and beneficial to a frail patient or to someone suffering the toxicities related to therapy can be difficult and requires special expertise. The sophistication in the field is improving and these practical hurdles will gradually be surmounted.

## 5. Conclusions

Muscle wasting is frequently associated with cancer and some cancer types such as pancreatic cancer and lung cancer have a particularly high association. Cancer-associated muscle loss is associated with a poor prognosis and susceptibility to treatment toxicities. Evidence is accruing that the tumor is an important driver of muscle wasting, as multiple tumor-derived pro-sarcopenic factors have been documented in animals as well as humans. These mediators act by multiple mechanisms that are known to be integral to the pathogenesis of muscle wasting, including systemic inflammation, systemic and local metabolic derangements and skeletal muscle proteolysis. An improved understanding of tumor-derived factors that drive muscle wasting may help to identify better therapies to improve the general health of the cancer patient. One possibility that must be considered is that the mediators driving loss of muscle may differ between tumor types or even between individuals. If this is the case, then therapeutic trials will need to take this diversity into consideration.
